# Effect of plastic deformation on the hydrogen embrittlement of ferritic high strength steel

**DOI:** 10.1038/s41529-025-00592-9

**Published:** 2025-05-02

**Authors:** Tim Boot, Pascal Kömmelt, Ruud W. A. Hendrikx, Amarante J. Böttger, Vera Popovich

**Affiliations:** 1 Department of Materials Science & Engineering, Delft University of Technology (TU Delft), Mekelweg 2, Delft, The Netherlands; 2 Research & Development, Tata Steel, PO Box 10000, IJmuiden, The Netherlands

**Keywords:** Metals and alloys, Mechanical engineering, Scanning electron microscopy, Characterization and analytical techniques

## Abstract

The effect of hydrogen charging during plastic deformation was investigated on a ferritic steel containing TiC nano-precipitates. Specimens were subjected to a slow strain rate tensile test (SSRT) up to 0, 1, or 3% plastic engineering strain, held until a total duration of 2 h to saturate with hydrogen, then fast fractured. The specimens pre-strained elastically absorbed 2.36 wppm of hydrogen, which increased to 3.69 wppm for 3% plastic strain. Only 0.72 wppm is stored in non-dislocation traps such as precipitates, grain boundaries, and lattice sites, which makes dislocations the main contributor to hydrogen trapping. The increased hydrogen uptake did not lead to a decrease in fracture strain, which remained between 6 and 10% for all pre-strains, compared to 60% for full SSRT tests that were charged for a shorter time. This research highlights the necessity of high plastic strains and the presence of hydrogen in the environment during crack growth to cause HE in ductile steels.

## Introduction

The automotive industry is a considerable contributor to global CO_2_ emissions, of which a large part is correlated to vehicle weight^1,2^. Reducing vehicle weight will therefore lead to CO_2_ reductions, but this has to be done without compromising passenger safety. For this reason, the industry has been at the forefront of using advanced high-strength steels (AHSS), which allow for critical parts in the vehicle to be manufactured from thinner material while retaining good mechanical properties to guarantee safety^3,4^. Good formability of these steels is required to obtain the complex geometries of structural car parts. Many high strength steels contain microstructural features that cause HE by attracting hydrogen which causes a reduction in mechanical properties and potential sudden fracture^5–10^. Examples of these are hard phases, interfaces or regions of stress and/or strain concentrations^11–14^. Accumulation in these areas is why diffusible hydrogen is the primary cause of HE^14–20^. One solution in automotive steels is the use of nano-carbide precipitates in a ferritic matrix to realise steels with a high strength as well as ductility without relying on large amounts of alloying elements. An added benefit of using nano-carbides is that they provide a mechanism for increasing resistance to hydrogen embrittlement (HE) by providing strong hydrogen trapping sites, without compromising on overall steel strength^12,15,17,18,21–25^.

The effects of precipitate type and size on HE were the focus of our previous research^11,26^. It was shown that large incoherent carbides provide strong hydrogen traps that do not cause HE, but that these traps cannot be charged at room temperature. The best performance was found in the case where nano-sized precipitates were present only in the grain interior^11^. In this case, HE was minimal and the only loss in ductility was detected after the onset of necking, whereas the material behaviour was unchanged at low strain. Park et al. find similar results in a study where pre-strained specimens were subjected to slow strain rate tensile (SSRT) tests in hydrogen, and their specimens show accelerated fracture only after the point of maximum stress^27^. HE was found to be most significant for specimens that had undergone strain hardening before being charged with hydrogen. Their results are supported by Li et al., who subject U-bent specimens to an acidic environment in a static test to observe the duration until fracture^28^. They find that fracture only happens in specimens strained until near the bending limit. Embrittlement in these studies is only seen in samples that are strained to the load limit. Takai et al. investigated the effect of straining during hydrogen charging, then unloading and annealing out the hydrogen before loading again to fracture^29^. They found that specimens that were strain hardened in hydrogen, but fractured without hydrogen, still showed a reduction in ductility compared to specimens only strained in air. Hydrogen charging during strain hardening is thus suggested to induce damage in the microstructure that reduces its total ductility regardless of whether hydrogen is being charged at the load limit. The discrepancy between these different studies raises the question whether the phenomenon of HE is only present at high plastic strains or whether it can be affected by hydrogen charged at low plastic strains.

It is known that straining during hydrogen charging increases hydrogen uptake. An increase in plastic deformation has the effect of increased hydrogen absorption since the defects that are created, such as dislocations and vacancies, provide hydrogen trap sites^29–32^. Moreover, the presence of hydrogen during straining can also increase the creation of defects, which further aggravates HE. This is explained in the Hydrogen Enhanced Strain Induced Vacancy (HESIV) model proposed by Nagumo^33,34^. Drexler et al. even found a hydrogen uptake of 9.09 wppm after straining a DP600 steel during hydrogen charging versus only 3.48 wppm after straining in air^30^. It can therefore be expected that straining during hydrogen charging affects HE since it induces a higher hydrogen absorption, although the local hydrogen concentration has been found to decrease temporarily upon the onset of dislocation motion by Fukunaga^35^.

This work studies the effect of only the hydrogen that is absorbed during strain hardening to answer whether or not straining up to the load limit is required to induce HE. The approach is to strain a ferritic high-strength steel to several degrees of plastic strain during an SSRT test and charge it with hydrogen until saturation. The hydrogen uptake is measured using Thermal Desorption Spectroscopy (TDS), before specimens are fast fractured to inhibit any further uptake of hydrogen at high plastic strains. The results are compared to both specimens strained in air and specimens strained in SSRT while hydrogen charging until fracture, and the hydrogen is correlated to deformation by both X-ray Diffraction (XRD) and Electron Backscattering Diffraction (EBSD) measurements to obtain a comprehensive understanding of the fracture mechanisms.

## Results

### Hydrogen absorption

**Fig. 1:**
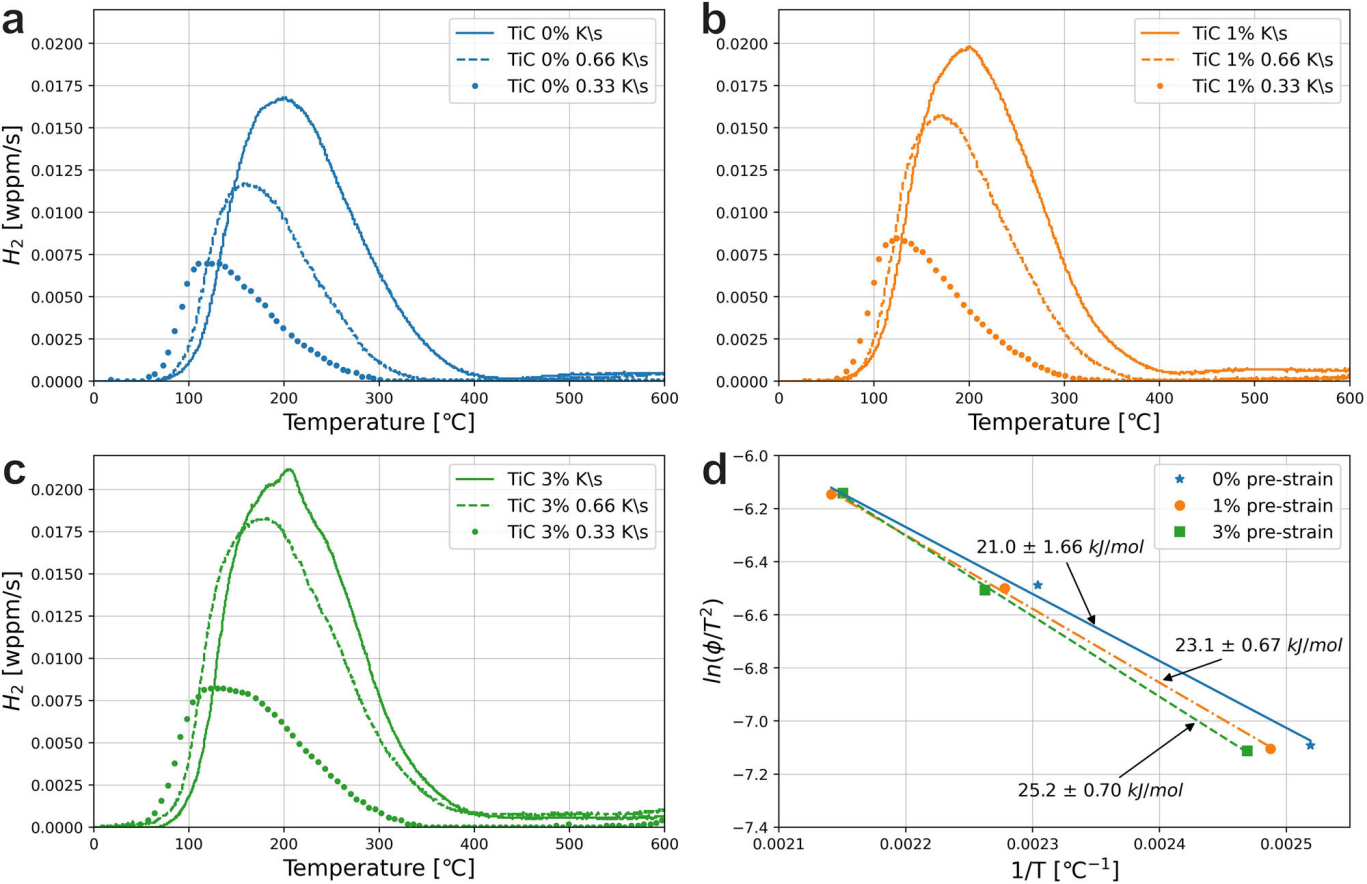
Hydrogen desorption and Kissinger curves. **a** 0%, **b** 1%, and **c** 3% plastic strain as well as **d** the Kissinger plot of the activation energies *E*_*A*_ for all pre-strains.

Table 1 contains an overview of the amount of absorbed hydrogen in each specimen after the charging and holding time of 2 h. The amount of H absorbed by the unstrained benchmark specimens was measured in our previous research under identical charging conditions and was found to be 2.18 ± 0.28 wppm^26^. It should be noted that the incoherent precipitates that are also present in the steel trap hydrogen in stronger sites that show desorption peaks at higher temperatures. These were, however, shown not to be further charged by the method of charging in this work, and they do not influence the mechanical behaviour of the steel^11,26^. Higher temperatures were therefore left out of the TDS measurements for this work. The hydrogen content found for the 0% pre-strained specimens is 2.36 ± 0.17 wppm, which is within the spread of that of the benchmarks. In contrast, the absorbed hydrogen content rises significantly upon the application of plastic pre-strain. The 1% and 3% plastically pre-strained specimens were found to absorb 3.00 ± 0.11 and 3.69 ± 0.26 wppm, respectively. The differences in hydrogen absorption between elastic and plastic pre-strain show the relative importance of dislocations as hydrogen trapping sites. The desorption curves for all specimens have been shown in Fig. 1. Skewed Gaussian peaks were fit to the measurement data represented in the figure, of which the maximum peak temperatures were used to calculate the activation energy of desorbed hydrogen in the peak. Table 1 shows them to be 21.0, 23.1, and 25.2 kJ/mol for increasing plastic pre-strain. The activation energy increases with plastic strain, but the extra hydrogen that is absorbed is stored in the same type of trap, namely the dislocation. Since trap occupancy in dislocations increases significantly, the increase in calculated activation energy can be understood to be an effect of re-trapping of hydrogen by dislocations before final desorption, which occurs, lowering the effective diffusivity of hydrogen throughout the steel^31,36^. The actual activation energy of a dislocation, however, would not change.

**Table 1:** Hydrogen absorption and mechanical behaviour properties of all tested conditions

	H content [wppm]	*E*_A_ [kJ/mol]	Fracture strain [%]	HEI [%]
Benchmark	2.18 ± 0.28	-	14.2 ± 0.47	-
0% Pre-strain	2.36 ± 0.17	21.0 ± 1.66	12.9 ± 2.08	9.2 ± 18.35
1% Pre-strain	3.00 ± 0.11	23.1 ± 0.67	13.2 ± 1.36	6.6 ± 13.13
3% Pre-strain	3.69 ± 0.26	25.2 ± 0.70	12.8 ± 2.01	9.5 ± 17.83
SSRT	3.48	-		59.8 ± 4.02

Errors are standard deviations.

**Fig. 2:**
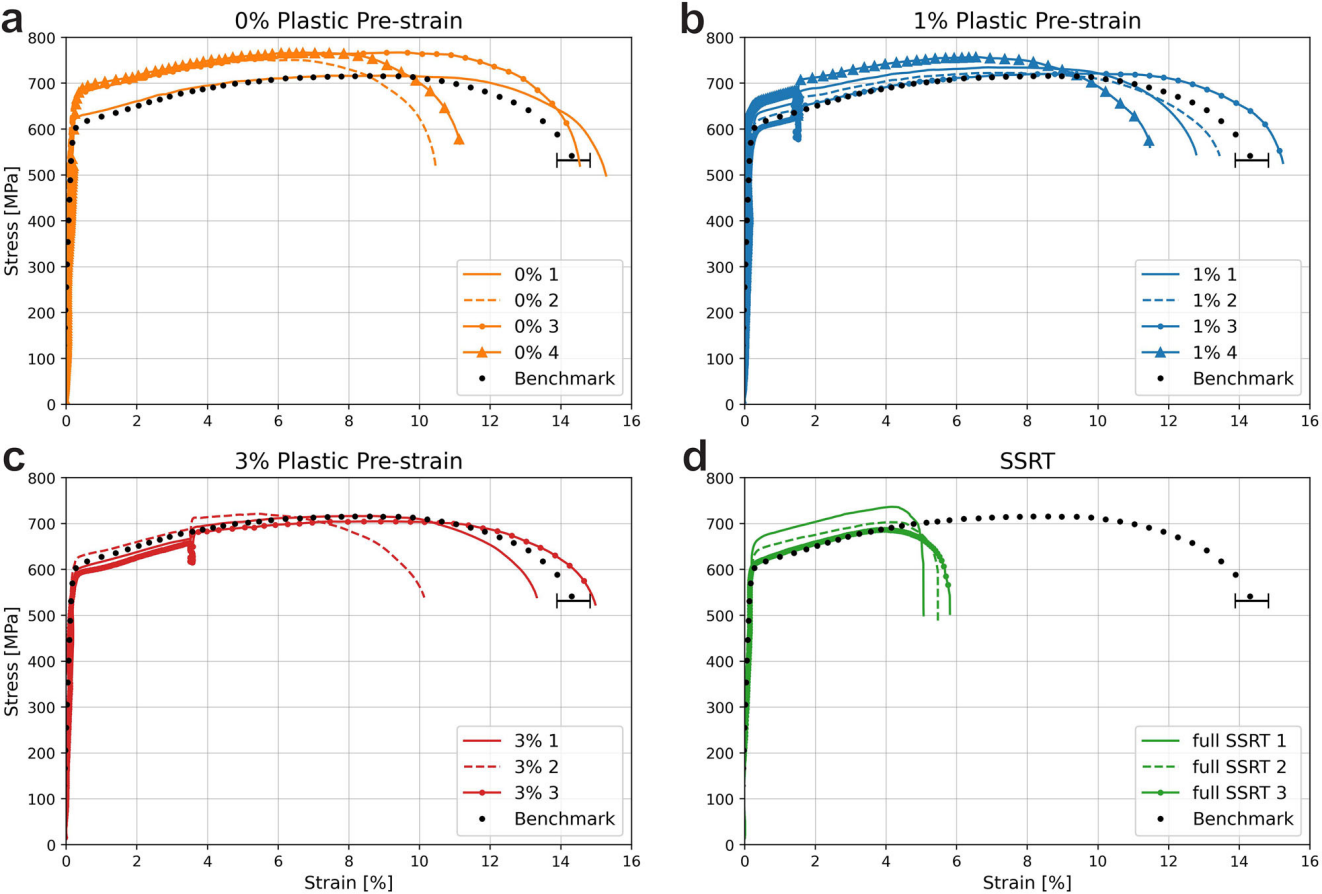
Strain curves for all specimens. **a** 0%, **b** 1%, and **c** 3% pre-strain, and **d** full SSRT curves. All tests except for benchmarks were performed during hydrogen charging. The error bar for the benchmark tests indicates the standard deviation of the final fracture strain.

Observing the mechanical behaviour in Fig. 2, it is evident that the increase in hydrogen uptake with increasing plastic pre-strain is not directly related to earlier final fracture. A trend is visible in the tensile curves, showing that, regardless of the amount of pre-strain, most tensile samples are not significantly more brittle than the uncharged benchmarks. Some individual samples are embrittled, but the degree of embrittlement is similar between specimens with different amounts of pre-strain or hydrogen uptake. The average HEI for the specimens was calculated to be 9.2%, 6.6%, and 9.5% for the 0%, 1%, and 3% pre-strained specimens, respectively. On the other hand, the SSRT specimens are consistently embrittled and fail around a strain of 5% even though they have been exposed to the charging environment for approximately 1 h, which is their time to fracture. A fractured SSRT specimen was measured in the TDS shortly after fracture and was found to have a hydrogen content of 3.48 wppm, which is within the spread of samples strained to only 3% after 2 h of charging. It therefore seems to have absorbed hydrogen more quickly. However, the strain after fracture is not homogeneous, so hydrogen is most likely segregated to regions of high ductility. Therefore, no conclusive quantitative statements can be made about the hydrogen concentration in the fracture specimen. The SSRT specimens are severely embrittled and have an HEI of 60%.

### Measurements of plastic deformation

**Fig. 3:**
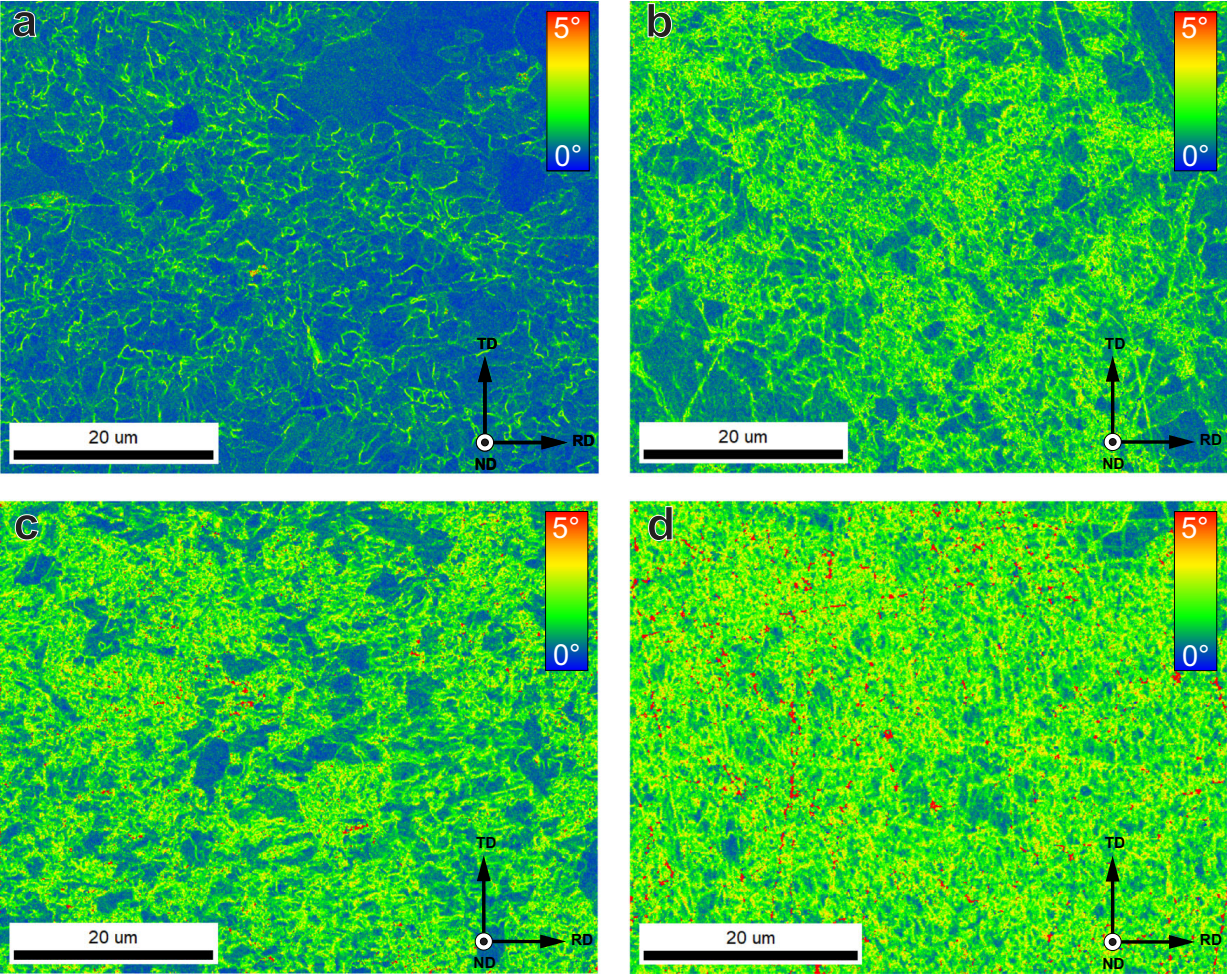
Kernel average misorientation maps. Specimens strained at **a** 0%, **b** 1%, **c** 3% plastic strain, and **d** in the necked area of a fractured SSRT sample.

Other than hydrogen content, for specimens at pre-strains of 0, 1, and 3%, the dislocation densities were determined through XRD and the KAM through EBSD. The necking area of an SSRT specimen was also characterised for both to provide data for the microstructure near the fracture in hydrogen. The results are shown in Table 2 and an overview of the KAM measurements is shown in Fig. 3. Although KAM measurements only include information on the density of Geometrically Necessary Dislocations (GNDs), and measurements from XRD also include Statistically Stored Dislocations (SSDs), the relative number of GNDs has been shown to constitute the majority of all dislocations up to the point of necking under tensile loading conditions^37^. The two techniques can therefore be used comparatively in this strain regime. Since the dislocation density is never zero, the specimen strained below the yield point already has a dislocation density of 1.8 ± 0.3 ⋅ 10^14^m^−2^, which corresponds to an average KAM of 0.6°. Both values increase with strain, where the largest relative increase is found between 0 and 1% plastic strain. The maximum is found in the necking area where the dislocation density is 4.4 ± 0.7 ⋅ 10^14^m^−2^ and the average KAM is 1.4°. It should be noted that the XRD is a macroscopic characterisation technique with an analysed surface area of roughly 1 cm^2^. Since the necked region has strain heterogeneities, the measurement for the SSRT specimen therefore has a larger error than the other dislocation densities. The KAM measurement is much more localised and was therefore taken in an area close to the fracture surface. This, as such, gives a more localised indication of the deformation near the fracture surface of the specimen.

**Table 2:** Dislocation density obtained from XRD, and Kernel average misorientation obtained from EBSD for specimens at 0, 1, and 3% plastic strain, as well as in the necked area of an SSRT specimen

	Dislocation density [10^14^*m*^−2^]	KAM [°]
0% Plastic strain	1.8 ± 0.3	0.60 ± 0.30
1% Plastic strain	2.8 ± 0.4	1.02 ± 0.53
3% Plastic strain	3.3 ± 0.5	1.16 ± 0.59
SSRT	4.4 ± 0.7	1.40 ± 0.62

## Discussion

**Fig. 4:**
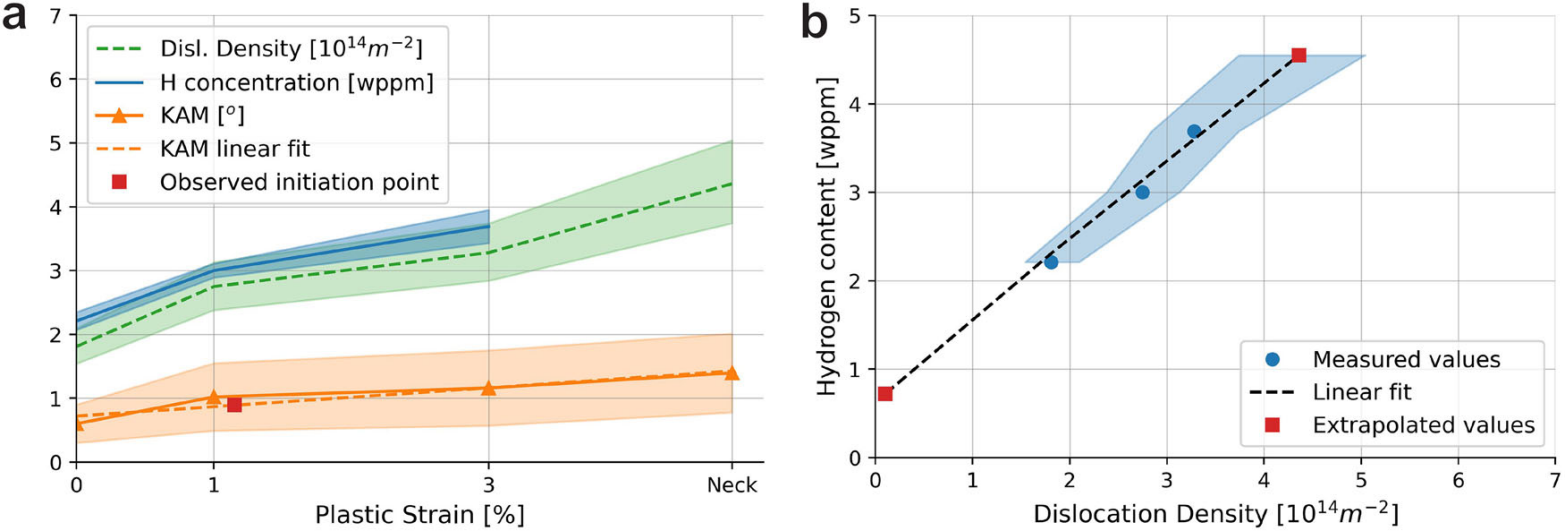
Correlations between dislocation density, hydrogen content, and kernel average misorientation. **a** The hydrogen content, dislocation density, and KAM as a function of the amount of Plastic Strain. Data for the `Neck' point on the *x*-axis was obtained on a necked area of an SSRT specimen. The average KAM value underneath brittle crack surfaces was indicated as the `Observed initiation point'. **b** The H content as a function of the Dislocation Density with extrapolated data for the ideal case of no dislocations, as well as the H content in the necked area of an SSRT specimen.

In this work, we attempt to quantify deformation of the microstructure by plotting the dislocation density, KAM values, and the hydrogen content as a function of plastic strain. Through measuring all three on specimens with known homogeneous macroscopic plastic strains for 0, 1, and 3%, a relation between them is established, and the effect of dislocations can be isolated. Figure 4a shows a graphical overview of the dislocation density, the KAM, and the hydrogen content of the specimens as a function of the plastic strain. The amount of plastic strain for the SSRT specimen is denoted as “Neck”, for which the location on the axis was determined as 4.8% by fitting it to the KAM measurements obtained for that area. Upon plastic straining, the amount of hydrogen traps increases in the form of newly generated dislocations. Figure 4b shows the hydrogen content as a function of the dislocation density to illustrate this effect. The data closely follows a linear regression, which has been extrapolated to both the average dislocation density in the neck region as well as a theoretical density of 0 for a dislocation-free polycrystal. The hydrogen content in the most deformed portion of the neck can be estimated to be 4.55 wppm if it were charged to saturation, but this was likely not the case. It is more interesting to consider the amount of absorbed hydrogen at a theoretical 0 dislocation density by extrapolating the curve to lower strain values. Although the actual dislocation density can never be zero due to the entropic effect, the number gives an indication of the amount of hydrogen that would be trapped in sites that are not dislocations. Examples of these sites could be lattice positions and grain boundaries, but also hydrogen that is stored in and on the interface of TiC nano-precipitates. From the linear extrapolation, this amount is calculated to be 0.72 wppm. This means that 1.64 wppm of hydrogen is stored in dislocations even in the 0% pre-strained specimens, which increases to 2.97 wppm for 3%. Even if precipitate traps were the only other traps other than dislocations, the hydrogen trapped in them would thus be less than a third of the amount of hydrogen that was absorbed in the 0% plastically strained specimen. The added hydrogen trapping capacity of the precipitates in this steel is therefore only of minor influence on the total capacity of the steel, especially when plastic deformation occurs. Furthermore, the traps that they present are considered diffusible with activation energies of less than 50 kJ/mol^11^. This means that while the TiC precipitates provide additional strength to the microstructure, their benefit to HE resistance could be limited. Perhaps their largest benefit is in retarding hydrogen diffusion throughout the microstructure, but it should be studied whether this makes up for the extra absorption of hydrogen.

**Fig. 5:**
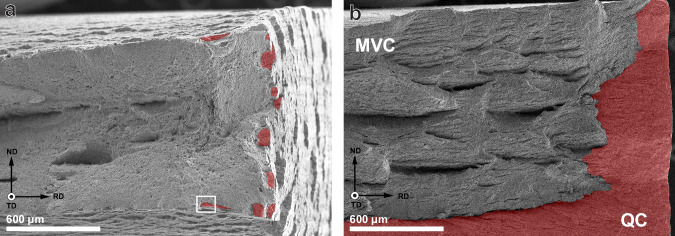
Fractographic overviews. Specimens **a** 3% and **b** SSRT, where brittle quasi-cleavage (QC) crack-initiated zones are indicated in red and microvoid coalescence (MVC) regions remain unmarked.

**Fig. 6:**
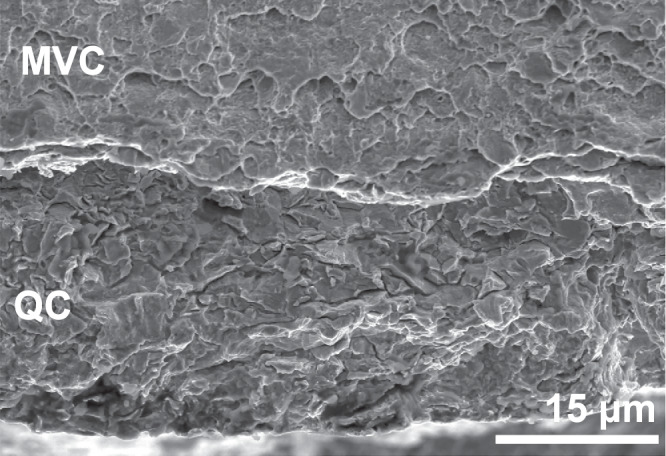
Close-up fracture surface. Transition zone from quasi-cleavage (QC) to microvoid coalescence (MVC) fracture in a 3% specimen, taken from the area indicated in Fig. 5a.

An important finding is that the increased hydrogen concentration caused by the increase in dislocation density from 2.36 ± 0.17 wppm to 3.69 ± 0.26 wppm does not increase the amount of embrittlement observed in the mechanical tests. Embrittlement was instead much larger in the SSRT specimens, although the hydrogen charging time for these specimens was the shortest. This means that, first of all, hydrogen charging to full saturation is not a prerequisite for HE. It also means that the steel can accommodate a higher hydrogen content than what is required to cause fracture, given that the plastic deformation is kept below a certain limit, in this case approximately 5%. This raises the question, however, of what happens at this level of deformation to cause the sudden fracture of the specimen. The fracture surfaces of post-mortem specimens can indicate the progression of a crack in the specimen. Figure 5a, b shows the fracture surfaces of the 3% 2 specimen and an SSRT specimen, respectively. As is also frequently observed in literature, brittle fracture initiation zones were discovered around the edges that were exposed to the hydrogen environment^27,28,38–40^. These zones correspond to the macroscopically brittle quasi-cleavage (QC) fracture mode and are indicated in red. The length of the brittle crack initiation zone corresponds to the width of the QC fracture zone on the fracture surface. This was measured to be between 30 and 50 μm for the pre-strained specimens, and it was found to be independent of the degree of plastic pre-straining. The SSRT specimens on the other hand, show a brittle zone of up to 500 μm, as can be seen in Fig. 5b. The fracture surface outside the brittle zones shown microvoid coalescence (MVC) type fracture that forms when the crack growth speed is too high to allow for continuous hydrogen diffusion to the crack tip. This can therefore only be formed during the fast fracture stage of the test. Since brittle fracture initiation occurs even in the 3% specimens, this is not the cause of failure that happens in the SSRT specimens. A higher magnification overview of the transition zone from QC to MVC fracture in a 3% specimen is given in Fig. 6.

**Fig. 7:**
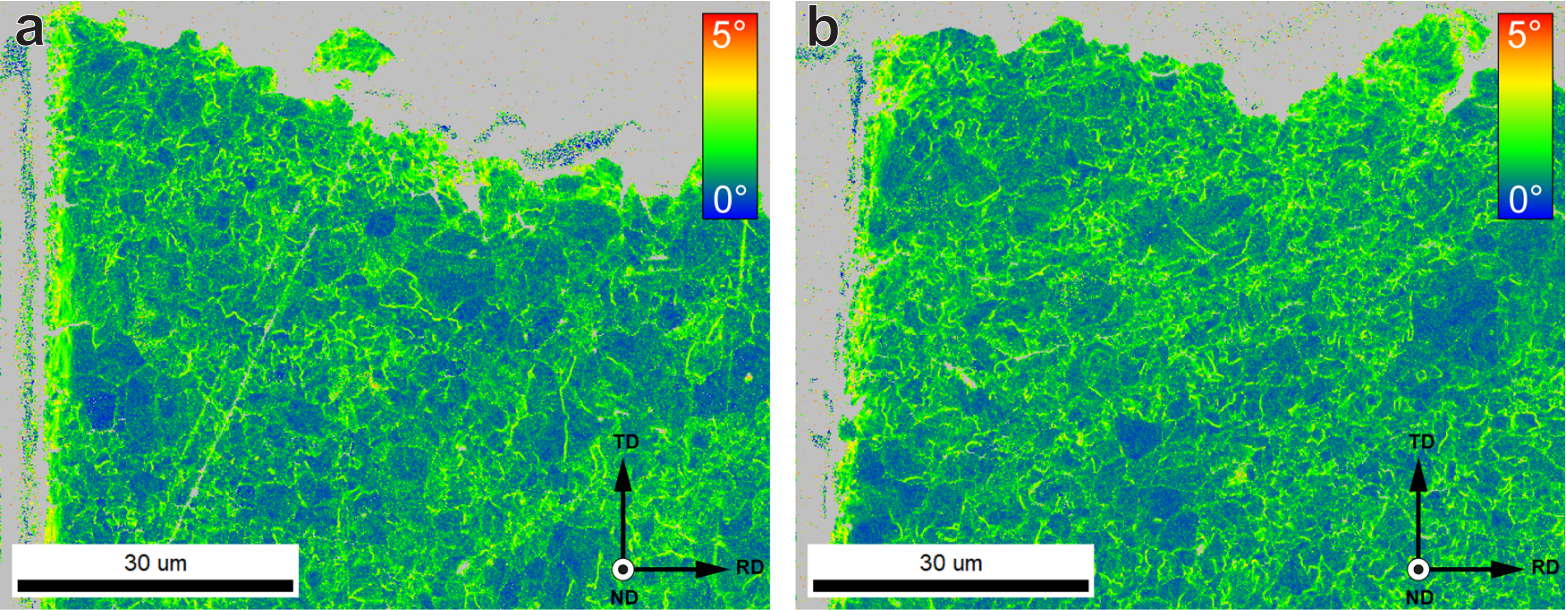
Post-mortem kernel average misorientation maps. Areas below a brittle initiation point on the fracture surface of **a** 0% and **b** SSRT specimens. Grey pixels were not included in the KAM measurement.

To find out at what point brittle initiation occurs, EBSD measurements were performed on cross-sections of selected specimens post-mortem. KAM maps of the sub-fracture surface microstructure beneath the brittle initiation zone were obtained. Figure 7a, b show two examples of these KAM maps. Figure 7b corresponds to the same sample shown in Fig. 5b, while 7a was taken from a 0% specimen exhibiting clear crack initiation on one side, providing a good study case to observe the amount of deformation required for brittle crack initiation. The average KAM values calculated beneath the crack surface are 0.89 ± 0.47°. This point is indicated in Fig. 4a to belong to a plastic deformation of just over 1% if fit to the regression. This value itself, however, is markedly below that of 1.02° obtained for a homogeneous plastic strain of 1%. This indicates that first crack initiation in the tests takes place early on during the deformation, when a homogeneous strain of 1% is reached. Evidently, these cracks do not propagate in the 3% specimens, but do in the SSRT specimens, where they grow large enough to cause macroscopic fracture.

**Fig. 8:**
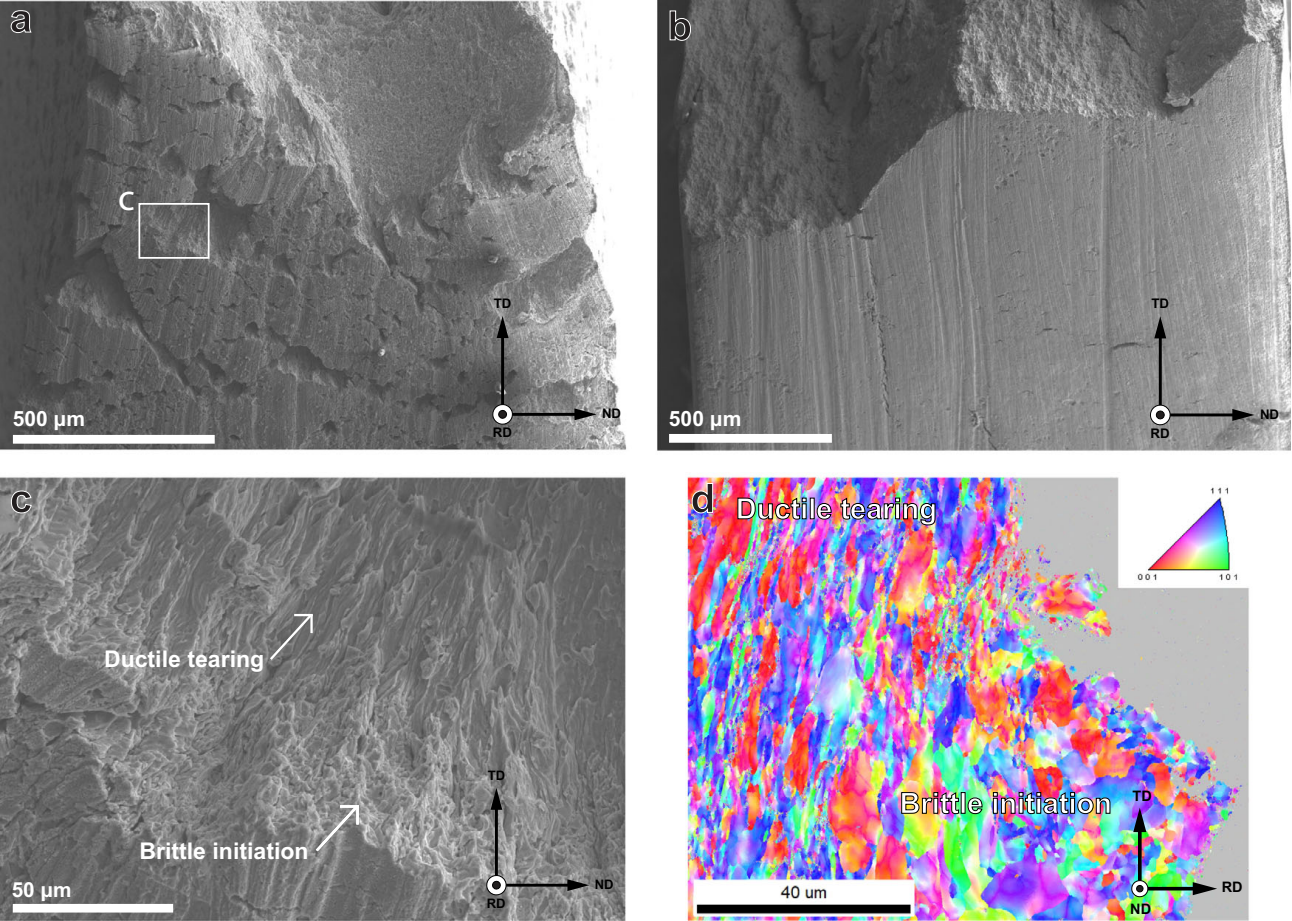
Tilted side views of post-mortem specimens. 45° tilted specimens **a** 3% and **b** SSRT. **c** shows a zoomed-in section of a crack in (**a**) to highlight the transition from brittle to ductile fracture. An Inverted Pole Figure (IPF) map of the cross-section of a similar transition is shown in (**d**), where the start of plastic deformation can be observed.

This finding is supported by Fig. 8a, b, which show the sides of the 3% 2 specimen and a SSRT specimen, respectively. The 3% pre-strained specimen shows a high amount of secondary cracks on the side surface, with significant crack mouth opening. In the SSRT specimen, on the other hand, the fracture is entirely localised to a single crack. Some secondary cracks can be observed, but they have not been opened. The opening of the secondary cracks in the 3% specimen is shown at a higher magnification in Fig. 8c. Here, the crack clearly displays a brittle fracture initiation zone that transitions into a region of ductile tearing. This is supported by an IPF map obtained from EBSD, shown in Fig. 8d, which shows the contrast between the relatively equiaxed grains beneath the brittle surface and the heavily deformed grains in the plastic region. These findings indicate that brittle cracks initiate at several locations during either the straining or hold time of the 3% specimen, but they arrest and are only opened during the further straining in the fast fracture stage of the test. Here, the strain rate is high enough that no more hydrogen is absorbed, diffusion towards the moving crack tip is too slow, and therefore the material retains its ductility. The SSRT specimens, on the other hand, were continuously strained at a low strain rate while being charged with hydrogen. This induces continuous crack growth where the crack tip can be supplied with hydrogen from the electrolyte, which causes brittle fracture even if the steel is not saturated with hydrogen. The extent of HE is therefore more correlated to the opening of the crack during hydrogen charging than the duration of charging itself and the resulting saturated hydrogen content.

The reason that the cracks initiated around 1% deformation do not propagate during the hold phase is unknown. A brittle crack that has initiated could be expected to grow under a constant opening in a hydrogen environment since new hydrogen is absorbed, and the stress concentration presented by the crack tip attracts hydrogen from the nearby bulk material. A possible reason for the crack arrest could be a small force drop during the hold phase of the tests as a result of the relaxation of the specimen and grips. This drop in force can be seen in Fig. 2 to occur for specimens strained above the yield point and could cause crack tip closure, depending on the degree of plastic deformation near the crack tip. The plastic deformation would be microscopic; however, given that the QC fracture surface is not formed from large-scale plastic deformation^41^.

This research shows that an increased internal hydrogen concentration does not necessarily increase HE. Instead, it is largely governed by the presence of hydrogen in the environment during crack initiation and propagation. The steel can absorb a significant amount of hydrogen without showing signs of embrittlement, provided it is not plastically deformed above a certain threshold. By varying the amount of plastic deformation, the trapping capacity of dislocations could be isolated from that of other features like precipitates, showing that the effect of dislocations on hydrogen trapping is much more significant than that of nano-precipitates. The following conclusions can be drawn from this work:

- Increasing load within the elastic region has little impact on the absorbed hydrogen content, which was 2.36 ± 0.17 wppm in the 0% pre-strained condition versus 2.18 ± 0.28 wppm in an undeformed specimen.
- Even small amounts of plastic deformation notably increase the H absorption in the steel from 3.00 ± 0.11 wppm for 1% up to 3.69 ± 0.26 wppm for 3% plastic strain.
- Up to 2.97 wppm of hydrogen is stored in dislocations, with only 0.72 wppm of hydrogen stored in other traps such as lattice sites, grain boundaries, and precipitates.
- Although beneficial for precipitation hardening mechanisms, precipitates do not play a dominant role in the hydrogen trapping capacity of ductile steels since significantly more hydrogen is stored in dislocations.
- Increased H absorption does not immediately lead to higher hydrogen embrittlement. All three levels of pre-straining resulted in 6 to 10% of ductility loss.
- Small brittle cracks begin to form around 1% global plastic strain, initiating on the outside surface of the steel that is in contact with the electrolyte and showing a Quasi-Cleavage fracture surface. These cracks only propagate in a brittle manner during continued slow-strain-rate tests. In such cases, the loss in ductility is 60%.

## Methods

### Heat treatment and microstructure

**Fig. 9:**
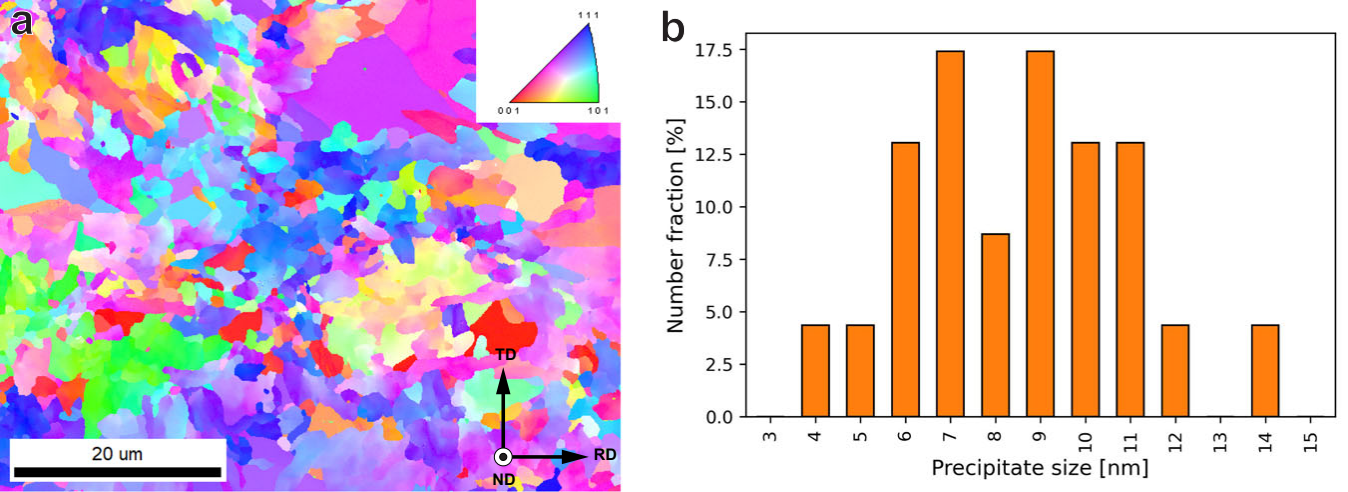
Overview of microstructure and precipitate sizes. **a** An IPF map of the microstructure of the undeformed steel, and **b** a histogram showing the distribution of semi-coherent precipitate sizes.

The steel used in this research was cast by Tata Steel in IJmuiden. The composition was measured on a PANalytical Axios Max Wavelength Dispersive X-ray fluorescence (WDXRF) instrument and is shown in Table 3. This composition was chosen to obtain a ferritic microstructure with finely dispersed TiC nano-precipitates for which he atomic ratio of Ti:C was kept as close to 1:1 as possible. The steel was heat treated by the same process as used in our previous work^11^, which consists of a heat treatment in pure N_2_ at 700 °C for 2 h. This time excludes a warm-up at 5 °C/min and a cooling-down that was furnace-limited at approximately 1 °C/min. Before the heat treatment, each specimen was sanded to a P1000 grit finish. The resulting microstructure can be observed in Fig. 9a, and b shows the histogram of precipitate sizes measured using TEM. The average grain area is 3.8 μm^2^, but extremes can be found on both the low and high ends. The sizes were therefore chosen to be listed in Table 4 according to the ASTM E1181 standard for randomly distributed grain sizes of a large range, the total range of observed grain areas was between 0.1 and 160 μm^2^.

**Table 3:** Summarised alloy contents of the TiC alloy in weight%

	C	Mn	Al	Si	Ti	Balance Fe
TiC	0.07	1.75	0.01	0.01	0.30	97.86

**Table 4:** Microstructural details for all heat treatment conditions

Hardness	Grain area [μm^2^]			Precipitate size [nm]			Precipitate
HV2	Low	Avg.	High	Low	Avg.	High	spacing
272 ± 3.1	0.1	3.8	160	2.8	8.6	290	
Semi-coherent nano-carbides							44 ± 23 nm
Homogeneously distributed incoherent > 100 nm carbides							3.0 ± 2.0 μm

A minimum of 100 precipitates were considered for the precipitate size and spacing measurements.

Precipitate sizes ($\sqrt{length\cdot width}$) and spacings were obtained using transmission electron microscopy (TEM),^11^, and are also listed in Table 4 by the same standard. Over 100 precipitates were measured manually on a Thermo Fisher Scientific^TM^ Cs corrected cubed Titan^TM^ TEM. The critical size for TiC precipitates to lose coherency with the iron matrix is 4.2 nm, which means that with an average size of 8.6 nm and an average spacing of 44 nm, virtually all precipitates in the steel are semi-coherent with the matrix^24^. Larger precipitates with sizes of >100 nm were also observed, but they are much more sparse, with an average spacing of 3 ± 2 μm. These were previously shown not to have any effect on fracture, even when charged with hydrogen^11^. The largest precipitate observed was 290 nm in size.

### In-situ multi-step tensile testing

**Fig. 10:**
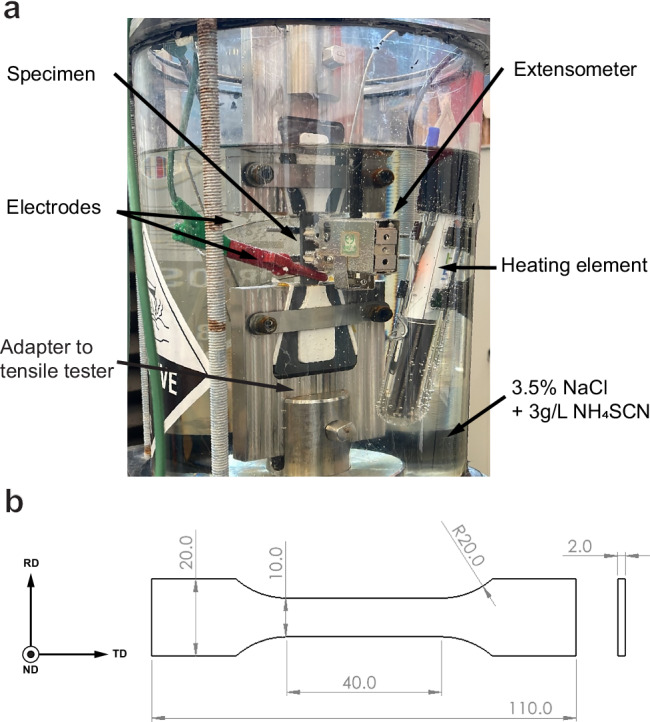
Overview of test equipment. **a** Overview of the in-situ hydrogen setup used for tensile testing and **b** the dimensions (in mm) of the tensile specimen geometry used in this study. Adapted from ref. ^26^.

**Fig. 11:**
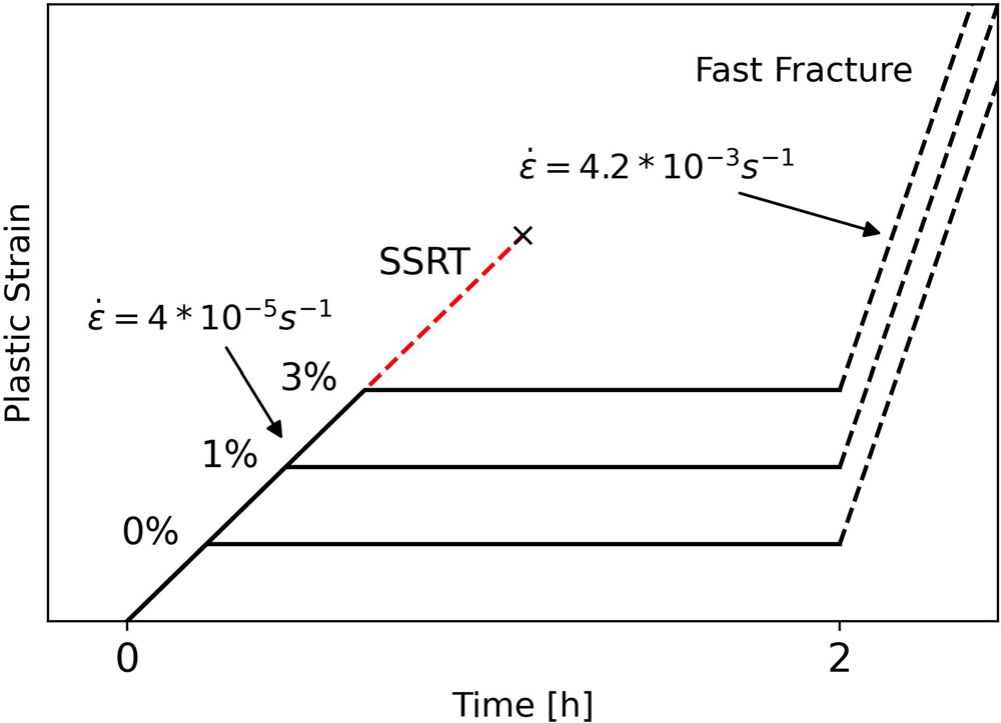
Overview of the test procedure. The entire test is performed during hydrogen charging, axes are not to scale.

All tests performed for this research were conducted in a custom-designed setup to allow for in-situ hydrogen charging during tensile loading. The setup and specimen geometry are shown in Fig. 10. The specimens are submerged in a 3.5% NaCl + 3 g/L ammonium thiocyanate (NH_4_SCN) solution for the duration of the test to allow for hydrogen charging. The charging is performed galvanostatically with a current density of 1 mA/cm^2^ for the entire duration of the test. A heating element is used to keep the basin at a constant temperature of 25 °C. Before testing, each specimen is sanded up to P1200 grit for a repeatable surface finish. The test itself consists of three steps, which are displayed in Fig. 11. The first step is a slow strain rate tensile (SSRT) test at a rate of 4 ⋅ 10^−5^s^−1^ up to a pre-determined level of either stress or strain. The crosshead position is then put on hold while charging is continued until the total test duration reaches 2 h, which was calculated as the saturation time for hydrogen charging of this steel in our previous work^26^. Finally, the specimen is subjected to a faster crosshead displacement of 10 mm/min until fracture, which corresponds to a strain rate of 4.2 ⋅ 10^−3^s^−1^. This is done to avoid additional hydrogen entry into the steel during the fracture phase of the tests, as well as to minimise the role of hydrogen diffusion during the fracture step. Any influence of hydrogen is thereby isolated to hydrogen absorbed during the first two steps of the test. The three threshold levels chosen were 0%, 1%, and 3% plastic strain, where 0% was set to 80% of the 0.2% proof yield strength of the uncharged specimens taken from previous research^26^. Some specimens were subjected to a one step SSRT tests which consisted of a strain rate of 4 ⋅ 10^−5^s^−1^ until fracture, which have been designated as SSRT. Other than that, benchmark specimens were tested at a similar strain rate without any hydrogen charging. The extent of HE was calculated using the fracture strains as per Equation (1) and listed as the Hydrogen Embrittlement Index (HEI). Here ${\epsilon }_{f}^{Air}$ and ${\epsilon }_{f}^{{H}_{2}}$ designate the fracture strains of uncharged benchmark SSRT specimens and hydrogen-charged specimens, respectively. Immediately after fracture of each specimen, both fractured halves were removed, rinsed with demiwater, dried with compressed air, and cleaned in the ultrasonic bath in isopropanol for 5 min. All tests were performed using a Zwick Z-100 universal tensile tester, and hydrogen charging was performed using a Bio-Logic VSP-300 potentiostat. Strain was measured using a submersible Epsilon 4030 extensometer.

1 $$HEI[ \% ]=\frac{{\epsilon }_{f}^{Air}-{\epsilon }_{f}^{{H}_{2}}}{{\epsilon }_{f}^{Air}}\cdot 100 \%$$

### Hydrogen analysis

Several specimens were removed from the test setup after the controlled hold step, and the gauge section of homogeneous strain was cut out. The gauge was then transferred to a Bruker G8 Galileo ONH thermal desorption analyser equipped with the IR07 furnace for accurate temperature ramping. Each strain condition was measured three times at heating rates of 1, 0.66, and 0.33 K/s from room temperature up to 600 °C. The amount of absorbed hydrogen was measured, and the peak temperatures were analysed using the simplified Kissinger equation^42^, which is shown in Equation (2). This equation allows for the determination of activation energies relating to individual peaks in the hydrogen desorption spectrum.

2 $$\frac{d\ln (\phi /{T}_{\max }^{2})}{d(1/{T}_{\max })}=-\frac{{E}_{A}}{R}$$

In this equation ${T}_{\max }$ is the peak temperature of the H desorption peak in K, *ϕ* the heating rate in K/s, *R* the universal gas constant 8.3415 J/molK and *E*_*A*_ the activation energy for hydrogen desorption of the specific hydrogen trap in kJ/mol. *E*_*A*_ can be derived from the slope by fitting $\ln (\phi /{T}_{\max }^{2})$ to $(1/{T}_{\max })$.

### Characterisation

Several characterisation methods were used to study the material in its undeformed and deformed states. Fractured specimens were prepared by cleaning in an ultrasonic bath with isopropanol. For electron backscattering diffraction (EBSD) images, the fracture surfaces were sectioned and embedded in a thermoplastic resin. The surface was polished up to a colloidal silica OPS finish of 0.04 μm, and the samples were cleaned in the ultrasonic bath afterwards. Both scanning electron microscopy (SEM) and EBSD were performed on a Thermo Fisher Scientific^TM^ Helios^TM^ G4 PFIB UXe SEM. Fractographic images were taken at an acceleration voltage of 20 kV and a probe current of 0.4 nA. EBSD maps were obtained at a voltage of 15 kV and a current of 6.4 nA to increase signal quality. The step size for all EBSD images shown in this work was 70 nm in order to obtain several indexed points even for the smallest grains, and a low cut-off angle of 5° was used for grain boundary identification. Selected EBSD specimens were used for X-ray Diffraction (XRD) measurements to obtain the dislocation density in all stages of deformation. The measurements were performed on a Bruker D8 Advance diffractometer using Co K*α* radiation, with a 2*θ* range of 40°–135°, a step size of 0.02°, and a counting time per step of 2 s. The modified Williamson-Hall method as used by Verma et al. was utilised to calculate the dislocation densities^43^. The smallest and largest crystallite sizes found were 75 nm and 260 nm for the necked area of a fractured SSRT specimen and an elastically deformed specimen, respectively. This means that the pixel size for the EBSD measurements was smaller than the crystallite size in all specimens. To negate the influence of the pixel size on the kernel average misorientation (KAM) measurements, the kernel included the 1st and 2nd nearest neighbour points, resulting in a kernel diameter of 350 nm, which is larger than the largest crystallite. All points in a kernel were used for the KAM calculation. The crystallite sizes were exclusively used as a guideline to choose the KAM kernel size. All other discussion of grain sizes uses the grain sizes as measured from EBSD.

## Data Availability

The datasets used and/or analysed during the current study are available from the corresponding author on reasonable request.
